# Maternal Oncological Disease and Congenital Pneumonia Are New Independent Risk Factors for Surfactant Requirements in Late Preterm Infants

**DOI:** 10.1111/apa.70250

**Published:** 2025-07-24

**Authors:** Simonetta Costa, Simona Fattore, Milena Tana, Giorgia Di Brina, Nicoletta Menzella, Paola Catalano, Benedetta De Santis, Chiara Barone, Giovanni Vento

**Affiliations:** ^1^ Neonatal Intensive Care Unit, Department of Woman and Child Health and Public Health Fondazione Policlinico Universitario Agostino Gemelli IRCCS Rome Italy; ^2^ Catholic University of Sacred Heart Rome Italy

**Keywords:** congenital pneumonia, maternal oncological disease, respiratory distress syndrome, risk factors, surfactant therapy

## Abstract

**Aim:**

The purpose of this study was to evaluate the incidence of respiratory distress syndrome (RDS) in late preterm (LPT) infants. We also aimed to identify the risk factors associated with surfactant requirement and the clinical outcomes associated with surfactant therapy.

**Methods:**

We retrospectively analysed data from a single‐centre neonatology unit at the Fondazione Policlinico Universitario Agostino Gemelli, IRCCS in Rome, Italy over a 4 year period. For each risk factor, the odds ratio (OR) and the 95% confidence interval (95% CI) were reported.

**Results:**

The incidence of RDS among 1335 LPT infants was 7.8%. The percentage of infants who required the surfactant was 3.4%. Gestational age (OR 0.541, 95% CI 0.367–0.796, *p* = 0.002), birth weight (OR 1.001, 95% CI 1.000–1.003; *p* = 0.037), 1 min Apgar score (OR 0.631, 95% CI 0.440–0.905, *p* = 0.012) and congenital pneumonia (OR 28.931, 95% CI 9.139–91.597, *p* < 0.001) were the neonatal risk factors associated with surfactant requirement. Oncological disease (OR 4.116, 95% CI 1.081–15.672, *p* = 0.038) was the maternal risk factor associated with surfactant requirement.

**Conclusions:**

Maternal oncological disease and congenital pneumonia emerged as new risk factors for surfactant requirement in LPT infants. These findings expanded the understanding of the mechanisms underlying RDS in LPT infants, moving beyond the known risk factors.

**Abbreviations**
CI: confidence interval
GA: gestational age
LPT: late preterm
OR: odds ratio
RDS: respiratory distress syndrome
SD: standard deviation

**Summary**

- This study aimed to evaluate the incidence of respiratory distress syndrome (RDS) in late preterm (LPT) infants and to identify the risk factors associated with surfactant requirement.
- We identified maternal oncological disease and congenital pneumonia as new independent risk factors for surfactant therapy in LPT infants.
- These results expand the understanding of the mechanisms underlying RDS, moving beyond the traditional risk factors such as gestational age and birth weight.

## 1 Introduction

Respiratory distress syndrome (RDS) due to surfactant deficiency is a common cause of morbidity in premature infants. The incidence of RDS is inversely proportional to gestational age (GA) [1, 2, 3, 4, 5]. Surfactant therapy mainly concerns 50% of infants born between 22 + 0 and 32 + 6 weeks/days of GA [6]. Therefore, most of the available studies on RDS focused on these categories of premature infants. The recommendations for surfactant therapy are primarily for infants with RDS born < 32 weeks of GA. The recommendations for the surfactant therapy in infants with RDS born late preterm (LPT) are borrowed from evidence obtained from infants born < 32 weeks of GA [7]. However, only 16% of the 15 million preterm births worldwide each year occur before 32 weeks of gestation, while over 84% occur later [8].

LPT infants, a worldwide increasing category of preterm infants born at 34 + 0–36 + 6 weeks/days of GA, constitute a unique group among all preterm infants. Risk factors leading to RDS may be different in this category compared to more preterm infants, as well as compared to more mature ones [9]. The large number of LPT infants contributes significantly to healthcare costs. For this reason, research efforts must be directed towards prevention strategies and the development of care pathways targeted at this category of preterm infants. However, knowledge of the risk factors for RDS and, mainly, for surfactant requirement is essential in order to direct research efforts towards specific prevention strategies.

The purpose of this study was to evaluate the incidence of RDS in a population of LPT infants admitted to a single‐centre neonatology unit. We also aimed to identify the independent risk factors associated with the need for surfactant and the clinical outcomes associated with surfactant therapy.

## 2 Methodology

### 2.1 Study Design, Setting and Population

This was a retrospective observational cohort study, performed at the neonatal intensive care unit of the Fondazione Policlinico Universitario Agostino Gemelli, IRCCS in Rome, Italy. It was carried out between 1 January 2020 and 31 December 2023.

All LPT infants with GA between 34 + 0 and 36 + 6 weeks/days were included in the study.

We excluded infants with congenital anomalies. Infants lacking the data necessary for the study's purposes and infants without parental informed consent for the use of anonymised data were also left out.

### 2.2 Primary and Secondary Outcomes

The primary outcome of the study was the incidence of RDS in our population of LPT infants. The secondary outcomes were to identify the independent risk factors associated with surfactant administration and to analyse the association between clinical outcomes and surfactant therapy.

### 2.3 Data Collection

Data relating to mode of delivery, multiple pregnancy, medically assisted procreation and antenatal corticosteroids administration were collected. Maternal oncological disease, pregnancy disorders such as gestational and pregestational diabetes, pregestational hypertension, pre‐eclampsia and the diagnosis of clinical chorioamnionitis were also registered.

Foetal growth restriction and foetal Doppler flow velocimetry abnormalities, intended as absent or reversed end diastolic flow velocity, were recorded.

GA, determined by the best obstetric estimate based on the first day of the last menstrual period, prenatal ultrasound and postnatal physical examination, was considered. Birth weight, birth weight *z*‐score, based on Intergrowth‐21 charts [10], gender and 1 and 5 min Apgar scores were also documented.

Lung ultrasound score, modality of surfactant administration and number of surfactant doses delivered were detailed. Congenital pneumonia, early onset sepsis, RDS, transient tachypnoea of the newborn, bronchopulmonary dysplasia [11], therapeutic hypothermia requirement, Grades 3–4 intraventricular haemorrhage [12], periventricular leukomalacia [13], persistent pulmonary hypertension, meconium aspiration syndrome, pneumothorax and death were recorded. Early‐onset sepsis was defined by a positive blood culture within the first 72 h of life. Diagnosis of congenital pneumonia was made on suggestive lung ultrasound findings. In patients who needed surfactant, congenital pneumonia was confirmed by a positive culture of the bronchoalveolar lavage fluid obtained just before the intubation–surfactant extubation procedure.

The duration of invasive and non‐invasive respiratory support, the duration of oxygen therapy and the length of hospital stay were also recorded.

RDS was defined based on clinical assessment of respiratory distress and inspired oxygen requirement in the presence of radiographic or ultrasound imaging compatible with RDS [7]. Infants who developed RDS were treated with 200 mg/kg of Curosurf (Chiesi Farmaceutici S.p.A., Parma, Italy). Surfactant was administered when the fraction of inspired oxygen > 0.30 on nasal continuous positive airway pressure ≥ 6 cm H_2_O and/or if lung ultrasound suggests surfactant deficiency [7]. A lung ultrasound score > 8 was considered suggestive of surfactant deficiency [14].

All data were collected from electronic clinical records.

### 2.4 Sample Size and Statistical Analysis

The sample size calculation was based on the observation that the incidence of RDS in LPT infants born in our center during 2023 was approximately 10%. To find the proportion of LPT infants with RDS, a sample of 139 LPT infants per year was needed [15]. This allowed us to find this proportion with a margin of error of 5% and a confidence interval of 95%. Since the study was planned for 4 years, at least 556 LPT infants were expected to be included in the study. The entire sample size allowed us to estimate the overall rate of RDS with a margin of error of 2.5%.

Demographic, clinical and laboratory characteristics of the sample were described by descriptive statistical techniques. Categorical variables were reported as absolute and relative frequencies (percentages). Quantitative variables were reported as means ± standard deviation (SD) if distributed as a normal, otherwise as median (interquartile range). We tested the assumption of normality in the distribution of continuous variables by Shapiro‐Wilks test. We compared categorical variables with chi‐square test or Fisher's exact test and continuous normal variables with Student's *t* test for independent samples or Mann–Whitney U test.

A logistic regression model was implemented to evaluate the association between surfactant administration and potential risk factors related to it. In addition, a further logistic model was fitted to evaluate the association between clinical outcomes and risk factors identified as statistically significant. Results with *p* < 0.05 were considered statistically significant.

Statistical analysis was performed using the statistical package for social science software (IBM Corporation, Armonk, NY, version 25).

### 2.5 Ethics

The study was carried out in compliance with the Declaration of Helsinki and does not contain any personal information that could lead to the identification of the patient. All data analysed were collected as part of routine diagnosis and treatment. Patient medical care was not set up for research purposes but was part of standard clinical procedure. The study protocol (Prot. ID 7055) was approved by the Institutional Review Board (CET Lazio Area 3). Written informed consent was obtained from the parents.

This study was reported following the ‘strengthening the reporting of observational studies in epidemiology’ reporting guideline [16].

## 3 Results

**FIGURE 1:**
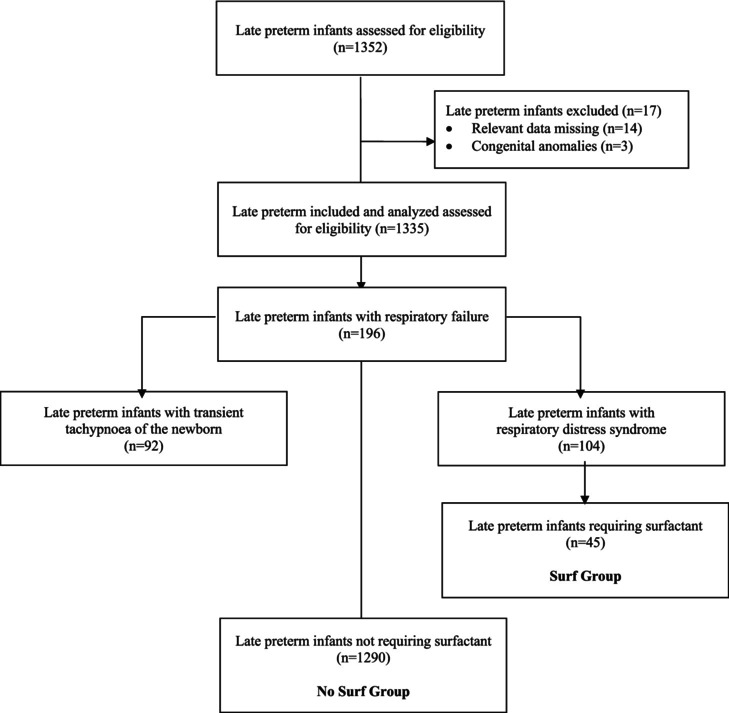
Consort flow diagram of the study.

During the study period, 1352 newborn infants admitted to the neonatology unit met the inclusion criteria. The newborn infants excluded were 17. Data relevant for the purpose of the study were missing in 14 neonates and congenital anomalies were present in three neonates. Finally, 1335 LPT infants were included and analysed. Of these, 196 newborn infants were admitted to the neonatal intensive care unit for respiratory failure. Among infants with respiratory failure, 92 were diagnosed as transient tachypnoea of the newborn and 104 as RDS. Overall, 45 newborns needed surfactant treatment (Surf Group) and 1290 did not (No Surf Group) (Figure 1).

Demographic and anamnestic data for the entire study population are shown in Table 1.

**TABLE 1:** Demographic, anamnestic and clinical data of the entire study population.

	*N* 1335
Gestational age, weeks	35.7 ± 0.8
Birth weight, grams	2375 ± 445
Birth weight *z*‐score	0.7 ± 1.1
Male gender	692 (51.9)
1 min Apgar score	9 (8–9)
5 min Apgar score	10 (9–10)
Vaginal delivery	402 (30.1)
Multiple pregnancy	524 (39.3)
Medically assisted procreation	157 (11.8)
Gestational diabetes treated with metformin	161 (12.1)
Gestational diabetes treated with insulin	177 (13.3)
Type 1 diabetes	15 (1.1)
Type 2 diabetes	27 (2.0)
Preeclampsia	135 (10.1)
Maternal oncological pathology	24 (1.8)
Clinical chorioamnionitis	8 (0.6)
Foetal growth restriction	177 (13.3)
Absent end diastolic/absent reversed end diastolic doppler velocimetry	111 (8.3)
Antenatal corticosteroids (any dose)	485 (36.4)
Antenatal corticosteroids (complete course)	373 (28.0)
Respiratory distress syndrome	104 (7.8)
Surfactant requirement	45 (3.4)
Transient tachypnoea of the newborn	92 (6.9)
Perinatal asphyxia requiring therapeutic hypothermia	9 (0.7)
Early onset sepsis	7 (0.5)
Congenital pneumonia	16 (1.2)
Meconium aspiration syndrome	1 (0.1)
Pneumothorax	10 (0.7)
Pulmonary haemorrhage	0
Persistent pulmonary hypertension of the newborn	3 (0.2)
Ventilator‐associated pneumonia	0
Bronchopulmonary dysplasia	0
Intraventricular haemorrhage > 2	2 (0.15)
Periventricular leukomalacia	4 (0.3)
Length of hospital stay	8.8 ± 21.9
Death	14 (1.0)

*Note:* Data are shown as number (percentage), mean ± SD, median (interquartile range).

The incidence of RDS in our population of LPT infants was 7.8%. The percentage of infants who required surfactant administration was 3.4%, that is, the 43.3% of LPT infants who developed RDS.

Most of the pregnancies were singleton (60.7%), and just over a third of the women had received at least one dose of antenatal corticosteroids. Caesarean deliveries occurred in 69.9% of the cases. Maternal complications, such as pregnancy disorders, oncological pathology and chorioamnionitis, were present in 41.0% of pregnancies (Table 1).

Table 2 shows demographic, anamnestic and clinical data for the two study groups. Infants who received surfactant were significantly younger than infants who did not. Despite lower GA, infants in the Surf Group were larger than infants in the No Surf Group.

**TABLE 2:** Demographic, anamnestic and clinical data of the study groups.

	No Surf Group *n* 1290	Surf Group *n* 45	*p*
Gestational age, weeks	35.7 ± 0.8	35.3 ± 0.9	0.003
Birth weight, grams	2366 ± 444	2601 ± 449	0.001
Birth weight *z*‐score	−0.57 ± 0.9	0.01 ± 0.2	0.003
Male gender	668 (51.8)	24 (53.3)	0.842
1 min Apgar score	9 (8–9)	8 (7–8)	< 0.001
5 min Apgar score	10 (9–10)	9 (8–9)	< 0.001
Vaginal delivery	388 (30.1)	14 (31.1)	0.960
Multiple pregnancy	518 (40.2)	6 (13.3)	< 0.001
Medically assisted procreation	153 (11.9)	4 (8.9)	0.810
Gestational diabetes treated with metformin	154 (11.9)	7 (15.6)	0.482
Gestational diabetes treated with insulin	168 (13.3)	9 (20.5)	0.178
Type 1 diabetes	13 (1.0)	2 (4.5)	0.088
Type 2 diabetes	27 (2.1)	0	1
Preeclampsia	132 (10.2)	3 (6.7)	0.615
Maternal oncological pathology	21 (1.6)	3 (6.7)	0.044
Clinical chorioamnionitis	8 (0.6)	0	1
Foetal growth restriction	174 (13.5)	3 (6.7)	0.260
Absent end diastolic/absent reversed end diastolic Doppler velocimetry	107 (8.3)	4 (8.9)	0.790
Antenatal corticosteroids (any dose)	469 (36.4)	16 (35.6)	0.910
Antenatal corticosteroids (complete course)	361 (28.0)	12 (26.7)	0.840
Perinatal asphyxia requiring therapeutic hypothermia	7 (0.5)	2 (4.4)	0.034
Early‐onset sepsis	7 (0.5)	0	1
Congenital pneumonia	9 (0.7)	7 (15.6)	< 0.001
Persistent pulmonary hypertension of the newborn	1 (0.1)	2 (4.4)	0.003
Meconium aspiration syndrome	1 (0.1)	0	1

*Note:* Data are shown as number (percentage), mean ± SD, median (interquartile range).

Surf Group had a significantly lower Apgar score at 1 and 5 min and underwent therapeutic hypothermia more frequently when compared to No Surf Group.

Infants treated with surfactant were more often born to single pregnancies and to women with oncological pathology. Moreover, infants in the Surf Group had a significantly higher occurrence of congenital pneumonia and persistent pulmonary hypertension of the newborn (Table 2).

Surf Group achieved a higher lung ultrasound score, needed higher levels of nasal continuous positive airway pressure and fraction of inspired oxygen than No Surf Group. Additionally, the durations of nasal continuous positive airway pressure and oxygen therapy requirement were longer in the Surf Group than in the No Surf Group. The duration of mechanical ventilation was similar between the two groups.

Surfactant was administered to 44 infants by intubation surfactant extubation technique and they were extubated within 30 min of the intubation. Only one infant received surfactant by less invasive surfactant administration technique. All 45 patients, including the only one who received surfactant via the less invasive surfactant administration procedure, showed immediate improved oxygenation. Multiple doses of surfactant were administered to seven infants, all with a diagnosis of congenital pneumonia (Table 3). The newborn infants, who received mechanical ventilation in the No Surf Group, required it at a later stage of life, mostly for surgical procedures (Table 3).

**TABLE 3:** Data relating to respiratory failure diagnosis and surfactant requirement.

	No Surf Group *n* 1290	Surf Group *n* 45	*p*
Respiratory support requirement	151 (11.7)	45 (100)	< 0.001
Nasal continuous positive airway pressure	133 (10.3)	45 (100)	< 0.001
Mechanical ventilation	18 (1.4)	7 (15.6)	< 0.001
Modality of surfactant delivery			
Intubation surfactant extubation	—	44 (97.8)	—
Less invasive surfactant administration		1 (2.2)	
Number of surfactant doses			
1 dose	—	38 (84.5)	—
2 doses		6 (13.3)	
3 doses		1 (2.2)	
Hours of life at first surfactant administration	—	15.1 ± 11.5	—
Oxygen therapy, *n*	151 (11.7)	45 (100)	< 0.001
Maximum of nasal continuous positive airway pressure requirement, cmH_2_O	6 ± 1	7 ± 1	< 0.001
Nasal continuous positive airway pressure > 6 cmH_2_O	26 (2.0)	27 (60.0)	< 0.001
Maximum fraction of inspired oxygen requirement, %	31 ± 10	43 ± 18	< 0.001
Lung ultrasound score at diagnosis	6 (4–6)	11 (6–12)	< 0.001
Nasal continuous positive airway pressure duration, hours	35.8 ± 43	86.6 ± 77	< 0.001
Mechanical ventilation, hours	61.3 ± 57.8	40.4 ± 48.5	0.370
Oxygen therapy duration, hours	21.1 ± 38.0	39.9 ± 46.5	0.019

*Note:* Data are shown as number (percentage), mean ± SD, median (interquartile range).

Pneumothorax and periventricular leukomalacia were the most frequent clinical outcomes among infants in the Surf Group, who also remained in hospital longer (Table 4).

**TABLE 4:** Clinical outcomes.

	No Surf Group *n* 1290	Surf Group *n* 45	*p*
Pneumothorax, *n*	8 (0.6)	2 (4.4)	0.042
Intraventricular haemorrhage > 2, *n*	1 (0.1)	1 (2.2)	0.066
Periventricular leukomalacia, *n*	2 (0.2)	2 (4.4)	0.006
Length of hospital stay, days	8 ± 20	22 ± 43	< 0.001
Death	14 (1.1)	0	1

We performed a multiple logistic regression analysis to identify the independent risk factors associated with surfactant requirement. GA, birth weight, Apgar score at 1 min, maternal oncological pathology and congenital pneumonia were significantly associated with surfactant requirement (Table 5).

**TABLE 5:** Multiple logistic regression analysis for surfactant requirement.

	OR	CI 95%	*p*
Gestational age	0.541	0.367–0.796	0.002
Birth weight	1.001	1.000–1.002	0.037
Birth weight *z*‐score	1.002	0.996–1.008	0.496
Multiple pregnancy	0.390	0.152–0.999	0.050
1 min Apgar score	0.631	0.440–0.905	0.012
5 min Apgar score	1.004	0.649–1.554	0.985
Therapeutic hypothermia	0.791	0.105–5.959	0.820
Maternal oncological pathology	4.116	1.081–15.672	0.038
Congenital pneumonia	28.931	9.139–91.587	< 0.001

Pneumothorax was associated with congenital pneumonia and not with surfactant administration in infants who received surfactant. Periventricular leukomalacia was associated with surfactant administration and with therapeutic hypothermia (Table 6).

**TABLE 6:** Multiple logistic regression analysis for clinical outcomes.

	OR	CI 95%	*p*
Outcome: Pneumothorax			
Surfactant requirement	0.493	0.041–5.910	0.576
Gestational age	0.709	0.219–2.304	0.568
Birth weight	0.998	0.996–1.001	0.210
1 min Apgar score	2.205	0.546–8.896	0.267
5 min Apgar score	0.587	0.117–2.941	0.517
Maternal oncological pathology	0.000	0.000	0.999
Therapeutic hypothermia	0.000	0.000	0.999
Congenital pneumonia	18.201	2.197–150.777	0.007
Persistent pulmonary hypertension of the newborn	0.000	0.000	0.999
Nasal continuous positive airway pressure > 6 cmH_2_O	4.708	0.470–47.215	0.188
Outcome: Periventricular leukomalacia			
Surfactant requirement	34.593	1.747–685.099	0.020
Gestational age	1.274	0.232–7.008	0.781
Birth weight	0.998	0.995–1.001	0.219
1 min Apgar score	0.949	0.313–2.877	0.926
5 min Apgar score	1.802	0.229–14.155	0.576
Maternal oncological pathology	0.000	0.000	0.998
Therapeutic hypothermia	482.611	5.420–42970.153	0.007
Congenital pneumonia	0.000	0.000	0.999
Persistent pulmonary hypertension of the newborn	0.000	0.000	0.999

## 4 Discussion

Our findings showed that the frequency of RDS was 7.8% in our population of LPT infants.

It is difficult to accurately compare our incidence with literature data. In the available studies, LPT infants were often considered together with early term infants or with moderately preterm infants. In these mixed populations, the incidence of RDS ranged between 8% and 10%, therefore, not far from ours [3, 17, 18].

The percentage of LPT infants who required surfactant therapy was 3.4% of the entire LPT infants cohort, nearly half of the infants who developed RDS. These data were consistent with those reported by Jackson et al., who found that, in their approximately 42 000 LPT infants with RDS, 45.6% required surfactant therapy [19].

The main risk factors for surfactant requirement were GA, birth weight, Apgar score at 1 min, congenital pneumonia and maternal oncological disease (Table 5).

Our analysis found that with increasing GA, the risk of requiring surfactant was reduced by 46%. This finding was consistent with that of a previous large‐scale cohort study that analysed the risk of RDS and surfactant requirement by individual GA [3]. The authors observed that 34 week old infants were 40 times more likely to develop RDS and 58 times more likely to develop RDS requiring surfactant. The risks for RDS and for RDS requiring surfactant decreased with each advancing week of GA until 38 weeks [3].

In our LPT infants, a higher birth weight was associated with an increased risk of surfactant requirement. This may be because the frequency of foetal growth restriction was twice as high in neonates who did not require surfactant compared to neonates who did. Although this difference was not statistically significant, it certainly determined a statistically significant difference in birth weight. Early studies suggested the possibility that growth restricted infants demonstrated accelerated lung maturation, advising that they may be at lower risk for RDS. This hypothesis came from the observation that the lecithin‐to‐sphingomyelin ratio, a marker of surfactant production, increased in the amniotic fluid of foetal growth restricted foetuses [20]. This may be due to the high plasma levels of cortisol present in these infants [21]. The randomised trial of foetal umbilical flow in Europe‐2 evaluated the benefits of antenatal corticosteroids for foetal lung maturation in pregnancies complicated by foetal growth restriction after 32 weeks of gestation [22]. The study results showed no benefit on short‐term outcomes, including neonatal RDS, in pregnancies that received antenatal corticosteroids compared to matched pregnancies that did not [22]. The higher incidence, even if not significant, of gestational diabetes in the Surf Group may be another explanation for birth weight as a risk factor for the surfactant requirement.

Two additional risk factors associated with the surfactant requirement in our LPT infants were the 1 min Apgar score and the presence of congenital pneumonia. Pneumonia, asphyxia and early onset sepsis were found to be responsible for neonatal acute respiratory distress syndrome in a study performed on 1005 neonates [23]. However, pneumonia has not been reported as a risk factor for RDS in LPT infants so far [24].

Neonatal respiratory failure resulting from hypoxia, present in both asphyxia and pneumonia, is due to a combination of primary surfactant deficiency and surfactant inactivation. Both of these conditions were reported to be the result of plasma proteins leaking into the airways from areas of epithelial breakdown and injury [25]. In an animal model, alveolar type II cells with positive surfactant type B protein staining were reduced after intrauterine acute ischaemic hypoxia exposure [26]. Messenger ribonucleic acid expression of surfactant proteins type A and B was also weak within the first 40 min after ischaemia [26]. Furthermore, hypoxia was associated with alterations of tight and gap junctions of the alveolar epithelium. These junctions alterations contributed to the development of lung oedema and impaired surfactant function [27].

Certainly, the decision to treat our infants with surfactant was based on individual clinical and ultrasound findings. The presence of pneumonia was diagnosed post hoc, that is, after obtaining the tracheal aspirate culture results. Although the diagnosis of pneumonia cannot be used to guide the decision to administer surfactant, this finding is useful to better understand the mechanisms underlying RDS. In particular, this finding showed how other factors, in addition to GA and birth weight, may be responsible for surfactant deficiency or inactivation.

Maternal oncological disease was a risk factor for surfactant requirement. Being born to a mother with cancer was reported as a risk factor for preterm birth and foetal growth restriction [28, 29]. Additionally, neonates from mothers with cancer were more frequently admitted to the neonatal intensive care unit [30]. Maternal malnutrition, stress and circulating cytokines may be harmful for placental development and foetal blood supply, with negative consequences for the fetus and the neonate.

We attempted to evaluate whether surfactant administration could be associated with specific clinical outcomes or could have a protective role. Considering both the low percentage of LPT infants who required surfactant therapy and the low percentage of adverse outcomes found, the analysis lacked statistical power. However, the association found between pneumothorax and pneumonia seemed to have a biological plausibility, as did the association between periventricular leukomalacia and therapeutic hypothermia for the reasons mentioned above.

## 5 Strengths and Limitations

This study had some strengths as well as some limitations that should be considered.

The strength of our study included the availability of data from a large sample of LPT infants from a single centre. This implied that in all infants the diagnosis of RDS and the administration of surfactant were based on standardised criteria. The standardised management avoided the slightly different criteria possibly used in other centers and allowed the generalisability of the results. The major limitation of the study was that, despite the large sample size, the adverse outcomes found were fortunately low. However, this prevented us from obtaining significant information regarding the effectiveness of the therapy in LPT infants in the event of a diagnosis of RDS.

## 6 Conclusion

In our study, the neonatal risk factors for surfactant requirement in LPT infants were low GA, higher birth weight, low 1 min Apgar score and congenital pneumonia. The maternal risk factor for surfactant requirement in LPT infants was the presence of oncological disease. These results expanded the understanding of the mechanisms underlying RDS in LPT infants, moving beyond the traditional risk factors such as gestational age and birth weight.

These findings were found in a large sample size of infants. Standardised diagnostic criteria for RDS and surfactant administration were employed. This approach ensured the reliability of the results and enhanced their generalisability, making the study a potential reference for the clinical management of LPT infants. Prospective studies are needed to obtain further information on the safety and efficacy of surfactant in this category of preterm infants.

## Author Contributions

S.C. conceptualised and designed the study, drafted the initial manuscript and critically reviewed and revised the manuscript. S.F. carried out the initial analyses and critically reviewed and revised the manuscript. G.D.B., N.M., P.C., B.D.S. and C.B. designed the data collection instruments and collected data. M.T. coordinated and supervised data collection and critically reviewed and revised the manuscript. G.V. critically reviewed and revised the manuscript for important intellectual content. All authors approved the final manuscript as submitted and agree to be accountable for all aspects of the work.

## Conflicts of Interest

The authors declare no conflicts of interest.

## Data Availability

The datasets generated during and/or analysed during the current study are available from the corresponding author on reasonable request.
